# Understanding the Association between Red Blood Cell Transfusion Utilization and Humanistic and Economic Burden in Patients with β-Thalassemia from the Patients’ Perspective

**DOI:** 10.3390/jcm12020414

**Published:** 2023-01-04

**Authors:** Russell L. Knoth, Shaloo Gupta, Kacper Perkowski, Halley Costantino, Brian Inyart, Lauren Ashka, Kelly Clapp

**Affiliations:** 1 Bristol Myers Squibb, 100 Nassau Park Blvd #300, Princeton, NJ 08540, USA; 2 Cerner Enviza, an Oracle Company, 51 Valley Stream Pkwy, Malvern, PA 19355, USA

**Keywords:** beta-thalassemia, direct costs, health-related quality of life, healthcare resource utilization, indirect costs, psychological burden, psychosocial burden

## Abstract

We assessed the humanistic and economic burden of chronic red blood cell (RBC) transfusions on patients with β-thalassemia. This cross-sectional, US-based study included adults (≥18 years) who self-reported a β-thalassemia physician diagnosis and had received ≥1 RBC transfusion in the past 6 months. The outcomes included the Functional Assessment of Cancer Therapy-Anemia (FACT-An), Patient Health Questionnaire-9, Generalized Anxiety Disorder-7, and ad hoc questions about treatment experience, side effects, direct/indirect costs, and psychological burden. Overall, 100 patients completed the survey, of whom 70% experienced “moderate” to “extremely high” burden due to RBC transfusions, 81% reported iron overload, 42% reported compromised social lives. The mean FACT-An score was 132 (higher score indicates better outcomes; 0–188). Mean scores were 33/52 for fatigue and 20/28 for anemia symptoms in the previous 7 days. Health-related quality of life (HRQoL) temporarily improved after RBC transfusion, although patients continued to experience mild-to-severe depression and anxiety, substantial direct costs, compromised employment, and suboptimal quality of life. Over 6 months, patients dedicated a mean of 173 h to transfusion requirements and incurred out-of-pocket costs of USD 2239 for transfusions and USD 896 for additional care costs. These findings highlight the need for new treatment options to improve patient HRQoL and economic outcomes.

## 1. Introduction

Beta-thalassemia (β-thalassemia) is a rare, inherited autosomal recessive blood disorder characterized by reduced or absent beta globin chain synthesis, resulting in reduced hemoglobin (Hb) levels, decreased red blood cell (RBC) production, and anemia [1,2]. The worldwide annual incidence of symptomatic cases is estimated to be 1 in 100,000 individuals [2]. Risk of β-thalassemia is most prevalent in the Mediterranean, Middle East, Africa, Asia, and India [1,2], and is classified as a rare condition in the United States of America (USA). However, the prevalence of β-thalassemia in the USA has increased by approximately 7.5% over the past five decades [3,4].

In patients with β-thalassemia, low Hb levels contribute to reduced oxygen levels in many parts of the body [5], and regular RBC transfusions are used to treat the resultant chronic anemia, supported by concomitant iron chelation therapy (ICT) to manage iron overload [3]. Patients’ ongoing dependence on RBC transfusions, compounded with treatment-related complications [6], has a substantial negative impact on the physical and mental health and overall health-related quality of life (HRQoL) of patients with β-thalassemia and their caregivers [7]. Advancements in the management of β-thalassemia have resulted in improvements in iron control and HRQoL over time; however, pain [8] and clinical complications including alloimmunization, iron overload, cardiac and liver disease, and endocrine and musculoskeletal disorders continue to impact patients’ care and lives [9]. Importantly, patients with transfusion-dependent β-thalassemia (TDT) report significantly poorer HRQoL compared to the US general population, with a meaningful impact on general health, physical and social functioning, and emotional well-being [9,10,11].

The burden of both clinical symptoms and a demanding treatment regimen for patients with TDT present challenges with treatment adherence [12,13,14] and work productivity [15], with subsequent increases in healthcare resource utilization (HCRU) and associated treatment costs [16]. Further research is needed to provide a more holistic understanding of the comprehensive burden of chronic RBC transfusions on patients’ lives, whether they are diagnosed with TDT or not, including psychological and psychosocial effects, employment decisions, direct costs, and the practical impact of ongoing RBC transfusion requirements on their health and living.

This study assessed the impact of β-thalassemia on HRQoL and work and activity impairment from the patient’s perspective. Secondary objectives were to characterize patients’ treatment experiences in terms of time commitments, psychological burden, burden of ICT and side effects, direct and indirect costs related to chronic transfusions, and caregiver burden.

## 2. Methods

### 2.1. Study Design and Data Source

This US-based, cross-sectional prospective study included 3 phases: an exploratory qualitative phase, cognitive pretest interviews, and a quantitative patient survey. Adult patients with β-thalassemia identified by the Cooley’s Anemia Foundation (CAF; www.thalassemia.org) between February 2021 and May 2021 were included. The exploratory qualitative interviews (*n* = 3) were conducted to dive deeply into the patient’s experiences and to ensure all relevant topics/concepts would be captured in the final quantitative survey. Cognitive pretest interviews (*n* = 3) were conducted to check for clarity and understanding of a web-based patient survey questionnaire.

Respondents to the 1-time, 30 min quantitative online survey conducted by CAF were recruited using a convenience sampling technique. Patients were recruited via email invitations or social media during a 3-week time period and responded to the survey via an email invitation. Informed consent was obtained before patients proceeded to the survey. Cerner Enviza administered the survey using personalized survey links provided to CAF, which were in turn provided as individual links to each patient to participate in the survey. 

This study was conducted in accordance with the International Society for Pharmacoepidemiology Guidelines for Good Pharmacoepidemiology Practices and in accordance with the ethical principles of the Declaration of Helsinki. The study was reviewed and granted exemption by the Pearl Institutional Review Board (IRB) (Indianapolis, IN, USA). 

### 2.2. Study Eligibility Criteria

Eligible patients were aged ≥18 years with a self-reported diagnosis of β-thalassemia (may include those with HbE-β-thalassemia) who had received ≥1 RBC transfusion in the past 6 months and could speak or read English (patients did not have to be considered TDT). Recruitment efforts targeted a minimum of 50% of patients who received ≥6 RBC transfusions in the previous 6 months. The study population for the quantitative survey was planned for ≥40 adult patients.

### 2.3. Study Assessments 

#### 2.3.1. Demographic Characteristics and Treatment History

Demographic variables included age, sex, race/ethnicity, marital status, annual household income, education, employment status, time since initial diagnosis, and prior treatment received for β-thalassemia. Patients also reported the number and frequency of RBC transfusions and the time since their last transfusion.

#### 2.3.2. HRQoL Outcomes

Patients reported their health status 1 week prior and within 1 week after their RBC transfusion based on recall of the time period. Response options included “very well”, “somewhat well”, “moderate”, “somewhat poor”, and “very poor”.

##### Patient Health Questionnaire-9 (PHQ-9)

HRQoL related to depression was reported using the PHQ-9 [17], which consists of a 9-item module with items scored from 0 to 3 [18]. Total scores of 5, 10, 15, and 20 represent cutoffs for categorization of mild, moderate, moderately severe, and severe depression, respectively [18]. 

##### Generalized Anxiety Disorder-7 (GAD-7)

Anxiety was assessed using the GAD-7, which is a 7-item scale where patients rate how they were impacted by different anxiety symptoms in the last 2 weeks, with each item scored from 0 to 3 (where anxiety was scored from 0 to 21 based on severity) [18,19]. Scores of 5, 10, and 15 represent cutoffs for categorization of mild, moderate, and severe anxiety, respectively [19]. 

##### Functional Assessment of Cancer Therapy-Anemia (FACT-An)

HRQoL was further assessed using the FACT-An questionnaire [20], which includes domains related to physical well-being, social/family well-being, emotional well-being, functional well-being, and additional concerns comprising 13 questions specific to fatigue and 7 questions related to feelings and experiences related to anemia [21]. Patients were asked to rate their experience related to each domain in the last 7 days on a 5-point scale ranging from 0 to 4, where higher scores are indicative of better HRQoL.

##### Work Productivity and Activity Impairment (WPAI)

The WPAI questionnaire was used to assess the effect of β-thalassemia-related health problems on the ability to work and perform regular activities [22]. The WPAI is a validated 6-item instrument evaluating 4 domains over the past 7 days: absenteeism (proportion of work time missed due to health), presenteeism (proportion of work time impaired due to health), overall work productivity loss (combination of absenteeism and presenteeism), and activity impairment (impairment of daily activities due to health). Only patients who reported being currently employed (working for pay) provided responses related to absenteeism, presenteeism, and overall work productivity loss.

#### 2.3.3. Time Burden

Patients reported the number of healthcare professionals (HCPs) who provided care, number of office visits with HCPs, laboratory/blood test visits, emergency room (ER) visits, and hospitalizations related to β-thalassemia over the past 6 months. The average time burden experienced by patients over the past 6 months was assessed for each type of visit: receiving blood transfusions (preparing for transfusion, waiting time prior to the transfusion, receiving the transfusion, and traveling to/from the transfusion location), scheduling appointments, getting insurance verification, calling about billing issues, and procuring and working with a social worker.

#### 2.3.4. Treatment Burden

Patients indicated their level of RBC transfusion-related burden on a scale of 1–7 as related to psychological burden, attitudes about treatment, fatigue, and impaired social life. The burden of associated side effects and time spent worrying about these side effects were also assessed. Concern regarding fertility issues was assessed for female respondents only.

#### 2.3.5. Burden of ICT and Comorbidities

The burden of ICT received during the past 6 months was assessed as related to the types of ICT, side effects experienced during ICT, and time spent worrying about side effects from ICT, including hives, fever, dizziness, severe allergic reaction, pain of bruising at injection site, lung injury, and heart failure. Overall comorbidities were also assessed using an adjusted Charlson Comorbidity Index (CCI) score [23]. 

#### 2.3.6. Direct and Indirect Costs

Direct medical costs to the patient associated with β-thalassemia treatment over the past 6 months included average out-of-pocket costs for HCP office visits, ER visits, hospitalizations, laboratory and blood tests, and RBC transfusions. Direct costs also included non-medical patient out-of-pocket costs associated with β-thalassemia treatment requirements, such as for childcare, transportation, parking/tolls, prescription medications, and over-the-counter medications. Indirect costs associated with β-thalassemia were also assessed based on responses to the WPAI questionnaire.

#### 2.3.7. Employment and Health Insurance

Patients indicated if and how their employment decisions were impacted by their β-thalassemia based on structured response options, including job choices related to health insurance plan, job security, proximity to treatment center, flexible working hours, non-physical work, other reasons, or if their β-thalassemia had no impact on their job choices. Patients with health insurance were also asked to indicate any burden related to health insurance coverage related to their β-thalassemia treatment according to the question: “How much of a burden, if any, are insurance verification and/or dealing with billing issues related to the blood transfusion process for the treatment of your β-thalassemia?”.

#### 2.3.8. Caregiver Burden

Patients indicated whether they had a caregiver, what their relationship was to the caregiver, whether they lived together, and the burden of their β-thalassemia on the caregiver. Caregiver burden questions were reported by the patient and were related to the amount of time in a typical week the caregiver dedicates to the patient, and how the patient’s β-thalassemia has impacted the caregiver’s time off from work or school, supportive activities, and health.

### 2.4. Statistical Analysis

Descriptive statistics were used to evaluate all study variables and outcomes, including counts and frequencies for categorical variables and means with standard deviations (SDs) for continuous variables. All analyses were conducted using Statistical Package for the Social Sciences version 25.0 (IBM Corp, Armonk, NY, USA) and R version 3.6.1 (https://www.R-project.org/) (accessed on 12 May 2021).

## 3. Results

### 3.1. Demographic Characteristics and Treatment History

A total of 134 patients with β-thalassemia were invited to participate, of whom 100 (75%) completed the survey and were included in the study. Thirty-four patients were excluded for the following reasons: did not access the link after receiving the email invitation (*n* = 27; 20%), had not received blood transfusions in the past 6 months (*n* = 6; 4%), and did not report being diagnosed with β-thalassemia by a physician (*n* = 1; 1%).

The mean (SD) age of included patients was 35.99 (10.36) years (Table 1). Approximately two-thirds were female (65%), and 42% were Asian or Pacific Islander. Annual household income was USD < 25,000 for approximately one-quarter of the study sample (27%), and USD ≥ 100,000 for one-third (33%). Approximately half of patients (55%) had an associate’s or bachelor’s degree. About half were married or living with a partner (46%), and more than half (63%) were employed. Mean (SD) time since the diagnosis of β-thalassemia was 34.40 (10.33) years. Nearly all patients (94%) had received ICT. Patients had received a mean (SD) of 9.62 (4.32) RBC transfusions in the past 6 months, with 70% of patients having received their last transfusion within 14 days of completing the survey. Approximately two-thirds (66%) of patients received transfusions more than once a month, and 34% received RBC transfusions monthly or every other month. 

### 3.2. HRQoL Outcomes

**Figure 1:**
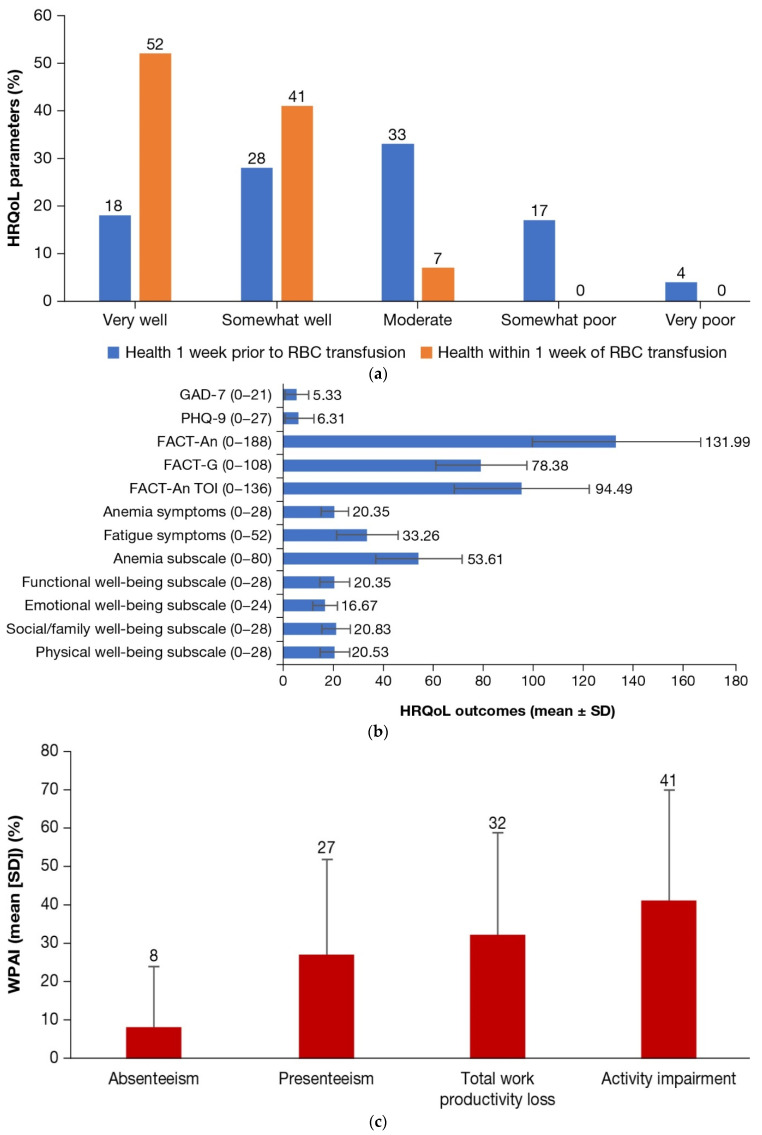
Health-related quality of life outcomes. (**a**) Health status 1 week prior and within 1 week of RBC transfusion; (**b**) HRQoL outcomes (mean FACT-An, PHQ-9, GAD-7 domains) (for subscales and FACT scales, higher scores imply better outcomes; for PHQ-9 and GAD-7, higher scores imply worse outcomes); (**c**) WPAI outcomes. Three individuals indicated that they are employed; however, they had not worked in the past 7 days. One of those individuals missed 40 h of work in the past week due to β-thalassemia. FACT: Functional Assessment of Cancer Therapy; FACT-An: FACT-Anemia; FACT-G: FACT-General; GAD-7: Generalized Anxiety Disorder-7; HRQoL: health-related quality of life; PHQ-9: Patient Health Questionnaire-9; RBC: red blood cell; SD: standard deviation; TOI: Trial Outcomes Index; WPAI: Work Productivity and Activity Impairment.

Approximately half (52%) of patients reported their health as very well 1 week after RBC transfusion versus 18% in the 1 week prior to RBC transfusion (Figure 1a). About half (52%) of patients had mild-to-severe depression and 53% experienced mild-to-severe anxiety in the previous 2 weeks (Table 2). The mean PHQ-9 and GAD-7 scores were 6.31 and 5.33, respectively (Figure 1b). The mean (SD) FACT-An total score on the scale of 0–188 was 131.99 (33.45). The mean scores for the FACT-An domains of physical well-being, social/family well-being, and functional well-being subscales ranged between 20 and 21, and for the emotional well-being subscale, the mean score was about 17. The mean (SD) score for the burden of anemia subscale was 53.61 (17.28) out of 80. Patients reported an average activity impairment of 40.70% (SD = 28.89%) and employed patients (n = 61) reported mean total work productivity loss of 31.79% (SD = 27.25%) due to β-thalassemia, as measured by the WPAI questionnaire (Figure 1c).

### 3.3. Time Burden

In the previous 6 months, 92% of patients had an office visit, 83% had a laboratory or blood test, 7% had an ER visit, 3% had a β-thalassemia-related hospitalization, and 10% were turned away from a transfusion due to a blood shortage (Table 1). During the process of a single transfusion, patients reported seeing an average of 5.11 (SD = 3.97) healthcare providers. Patients with an office visit in the past 6 months spent a mean of 3.99 (SD = 5.41) h in office visits (*n* = 92). Those with blood tests (*n* = 83) spent an average of 3.21 (SD = 2.85) h getting the blood tests, and those with ER visits (*n* = 7) spent a mean of 5.93 (SD = 3.45) h for ER visits. Three patients had a β-thalassemia–related hospitalization in the previous 6 months, with a mean length of stay of 2.17 (SD = 1.15) days. 

**Figure 2:**
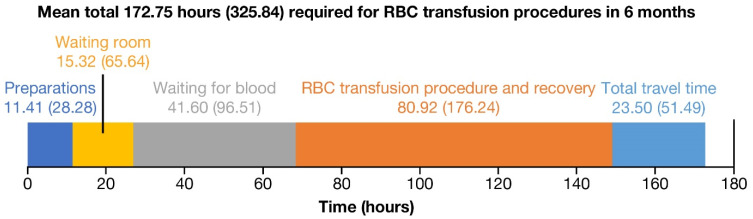
Mean (SD) hours spent for transfusion sessions over a 6-month period. RBC: red blood cell; SD: standard deviation.

Over a 6-month period, patients spent a mean of 172.75 (SD = 325.84) h for all procedures related to the transfusion process, approximately half of which (mean = 80.92, SD = 176.24 h) was comprised of the transfusion procedure and recovery time (Figure 2). Patients spent over 11 h getting blood drawn in the previous 6 months (mean = 11.41, SD = 28.28 h). Travel time (mean = 23.50, SD = 51.49 h) and wait times (mean = 56.9 h) comprised an additional 80.42 h over 6 months. Time spent scheduling appointments, managing billing/insurance issues and working with a social worker comprised 8.51 (SD = 11.41) h over 6 months (Table 3). 

### 3.4. Treatment Burden

**Figure 3:**
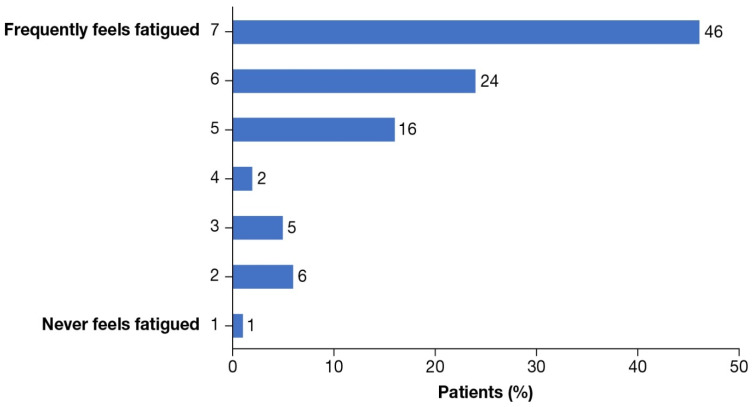
Patients reporting fatigue. Patients indicated the level of fatigue they feel leading up to their blood transfusion (7-point scale from “never feels fatigue” to “frequently feels fatigue”).

Nearly all (91%) patients reported that RBC transfusions resulted in psychological burden, of whom 20% reported a “high” burden and 14% reported an “extremely high” burden. Forty-one percent of the patients stated that their β-thalassemia treatment had become a routine part of their lives and 25% of patients considered their treatment routine a burden (Table 1). Of the patients, 71% wondered about life without β-thalassemia, 86% frequently felt fatigued leading up to a blood transfusion (Figure 3), and 42% claimed not being as social with friends/family due to the time needed for RBC transfusions (Supplementary Table S1). 

The most common side effects experienced during RBC transfusion were iron overload (81%), followed by rash/hives (74%), fatigue (61%), and pain/bruising at the transfusion site (52%). The other common side effects included fever and headache (39% each), severe allergic reactions and bloating (32% each), and endocrine dysfunction (29%). Nearly 57% of the female patients had concerns about fertility issues (Supplementary Table S1). 

**Figure 4:**
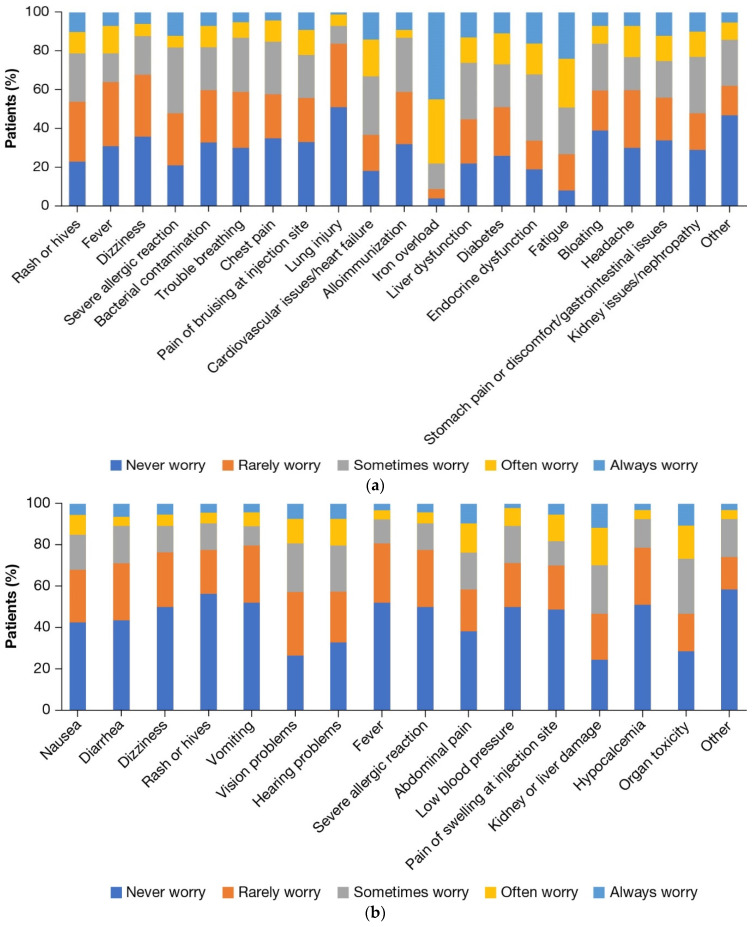
Psychological outcomes. (**a**) Frequency of worrying about transfusion side effects; (**b**) Frequency of worrying about ICT side effects. Patients indicated how frequently they worried about predefined ICT-related side effects in the past 6 months (5-point scale from “never worry” to “always worry”). ICT: iron chelation therapy.

In terms of transfusion side effects, patients reported spending the most time worrying about iron overload, fatigue, endocrine dysfunction, and cardiovascular issues (Figure 4a).

### 3.5. Burden of ICT and Comorbidities

Overall, 94% of the patients received ICT, of whom 93% received ICT orally and 19% received ≥1 type of ICT. Many patients reported frustration with their ICT treatment (52%) (Supplementary Table S2). The most commonly reported side effects of ICT were nausea (40%), diarrhea (37%), abdominal pain (35%), pain/swelling at the injection site (35%), and hearing problems (30%) (Supplementary Table S2). Patients worried most about potential ICT side effects related to organ toxicity and vision problems. Few patients (15%) worried about ICT-related nausea often or all the time, and the remainder never (43%), rarely (26%), or sometimes (17%) worried about ICT-related nausea (Figure 4b). 

The most commonly reported comorbidities were anemia (33%), osteoporosis (33%), and anxiety (18%). Most patients (76%) had a CCI of 0, and only 11% had a CCI score ≥3 (Supplementary Table S2).

### 3.6. Direct and Indirect Costs

The mean total out-of-pocket costs of care in the previous 6 months among all patients regardless of HCRU (excluding RBC transfusions) was USD 896.15 (SD = USD 3066.84), with an additional USD 2239.18 (SD = USD 5537.33) attributed to RBC transfusions (Table 4). Component costs were calculated for patients who reported associated HCRU/visits, including for office visits (mean = USD 555.23, SD = USD 1981.87 among those with ≥1 office visit, *n* = 92), ER visits (mean = USD 143.00, SD = USD 153.76 among those with ≥1 ER visit, n = 7), hospitalizations (mean = USD 41.67, SD = USD 52.04 among those with ≥1 hospitalization, *n* = 3), and laboratory/blood tests (mean = USD 450.70, SD = USD 1531.11 among those with ≥1 test, *n* = 83). Additional costs related to β-thalassemia treatment requirements in the previous 6 months comprised a mean total of USD 962.34 (SD = USD 2457.98). Contributing costs for patients who needed them included those for childcare (mean = USD 1780.00, SD = USD 2778.69, *n* = 9), transportation (mean = USD 178.83, SD = USD 177.64, *n* = 72), parking/tolls (USD 111.00, SD = USD 131.50, *n* = 54), prescription medications (mean = USD 791.59, SD = USD 2501.01, *n* = 59), over-the-counter (non-prescription) medications (mean = USD 157.50, SD = USD 192.06, *n* = 48), and other costs (mean = USD 885.00, SD = USD 1584.53, *n* = 8). 

### 3.7. Employment and Health Insurance

Approximately 83% of employed patients’ job decisions were affected by their β-thalassemia, with 59% of their employment choices related to the employer’s health insurance plan. Overall, 98% of patients reported having health insurance, most often a Managed Care Plan (57%) followed by Medicare or Medicaid (24% each). Health insurance covered most patients’ prescription medications (91%) (Table 1). Most patients (76%) reported at least “some burden” of insurance-related issues; only 24% reported no burden of insurance-related issues (Table 4). 

### 3.8. Caregiver Burden

A total of 13 patients reported having a caregiver (13%), of whom 46% reported a parental relationship with the caregiver, and 77% reported living with their caregiver. Caregivers most prominently assisted with transportation (77%), household duties (77%), picking up medications (62%), and/or assistance in treatment decisions (62%) (Supplementary Table S3). Approximately one-third (39%) of patients reported their caregivers to have “somewhat negatively impacted” or “very negatively impacted” physical health due to the patient’s β-thalassemia. Approximately half to one-third of caregivers were reported to have at least “somewhat negatively impacted” finances (54%), mental health (47%), and social health (31%). On average, caregivers were reported to spend 32.46 h per week helping the patient due to their β-thalassemia over the previous 6 months (Supplementary Table S3).

## 4. Discussion

This real-world observational study evaluated the humanistic and economic burden of chronic transfusions for patients with β-thalassemia in the USA. These findings demonstrated a comprehensive burden of chronic transfusions, including depression and anxiety, increased fatigue, compromised HRQoL, and notable psychological and psychosocial effects. Treatment for β-thalassemia was associated with a substantial time burden due to frequent RBC transfusions, only half of which was attributable to the clinical visit for the transfusion. The additional non-clinical time and cost requirements of β-thalassemia and an ongoing treatment regimen were considerable, both to patients and their caregivers. Most working patients considered their β-thalassemia and transfusion needs in their employment decisions, particularly as related to health insurance and job security, and had compromised work productivity, performance of daily activities, and substantial HCRU and associated costs. 

Although patients in this study reported that their health improved after the RBC transfusion, only half reported their health status as “very well” in the context of a notable prevalence of self-reported symptoms of depression and anxiety. Most patients in this study had RBC transfusions monthly or more often, which appeared to have a meaningful impact on the employed patients. Even though the mean age of the population was well within the typical working age, only two-thirds of patients reported being employed, though a direct connection to their β-thalassemia was not established in the survey. Compromised HRQoL was observed consistently across instruments and specific domains of living. Similarly, Cappellini et al. (2019) showed worse quality of life (QoL) among TDT patients compared with non-transfusion dependent β-thalassemia (NTDT) patients [10], and other studies have reported substantial emotional burden [24,25,26], depression and anxiety [27], and anemia and related symptoms (such as fatigue) [28,29], as having a detrimental impact on HRQoL and subsequent work productivity impairment in patients with TDT [15]. 

Patients in this study reported a substantial time burden related to receiving RBC transfusions, including travel and overall management of treatment requirements. Paramore et al. (2021) also reported a meaningful time burden related to transfusions and associated requirements such as travel and managing insurance issues [30]. The time burden of RBC transfusion requirements experienced by patients with β-thalassemia is similar to reports of the burden of health-related activities for other chronic diseases, such as diabetes mellitus [31]. These findings may be further explored and refined in a time and motion study that would provide greater detail and insight into the laborious requirements of RBC transfusion dependence. 

The side effect burden of RBC transfusions observed in this study showed particular concerns related to iron overload, rashes or hives, and fatigue. These results are consistent with prior studies that reported transfusion side effects such as iron overload, severe allergic reactions, endocrine dysfunction, cardiovascular issues [16], and fatigue due to the transfusion procedure [30]. Side effects due to ICT such as kidney or liver damage and organ toxicity were also of concern to patients receiving ICT and RBC transfusions, consistent with prior reports [32], which have also shown diarrhea, rash, and abdominal pain [33] to impact patients’ satisfaction with ICT. Careful monitoring of both iron toxicity and the effects of excessive chelation as well as appropriate dose adjustment, regular adherence to treatment regimens without interruption, and tailored ICT are essential in the management of iron overload and improved outcomes in thalassemia [6]. 

The majority of employed patients’ job decisions considered their β-thalassemia needs, especially those related to health insurance, job security, and flexibility. The average total out-of-pocket costs of care for patients in the previous 6 months were USD 896, with an additional USD 2239 spent on transfusions and USD 962 for associated costs outside of care. This is an approximate patient expense of USD 683/month which may be meaningful in the context of a population where 39% reported a salary USD < 50,000/year (gross USD 4200/month). In addition to the costs borne by patients, the majority of care-related costs are carried by the health insurance plan, which should be further investigated to quantify the costs of chronic RBC transfusions for this population. The high costs of transfusions [15,34] have been reported previously, highlighting the need for effective medical treatments that would decrease RBC transfusion dependence for patients and, in turn, would be likely to alleviate associated costs to employers and payers. 

Only 13% of patients in this study reported having a caregiver, though the sample size was relatively small and more independent patients may have been more likely to opt-in to participate in the survey. As such, patient-reported caregiver burden should be interpreted with caution, and future studies may consider soliciting responses directly from caregivers. Treating physicians should advise their patients [35] to seek social interaction and assistance from their caregivers and family to help manage treatment-related challenges [36]. Further work is needed to more fully understand the burden of β-thalassemia and a demanding treatment regimen on caregivers.

### Strength and Limitations 

Since β-thalassemia is considered a rare disease in the USA, the major strength of this study is giving voice to the experience of patients with β-thalassemia. There is otherwise a general scarcity of patient-reported outcomes related to TDT in the literature, especially concerning the practical requirements of regular RBC transfusions. The demographic and clinical characteristics of patients with β-thalassemia in the USA reported in this study may also help clinicians identify candidates for early screening. This study used an iterative survey development process to test and refine survey questions for clarity and relevance to this patient population. Findings from this study can help to inform future research programs and serve the needs of patients, caregivers, and clinicians. 

This study should also be interpreted in the context of certain inherent limitations. The respondents provided self-reported physician diagnosis of β-thalassemia and caregiver burden, which may have introduced measurement error. The diagnosis, treatment history, and other clinical outcomes were not confirmed by a physician or chart review. Patients were asked to recall information and experiences over the previous 7 days and 6 months, which may have been prone to recall bias, as in most survey studies. The study sample was recruited through CAF, an advocacy group that strives to increase the life expectancy and QoL of patients with thalassemia by informing them and their family members about the disorder and its challenges. Hence, recruited patients who were engaged with or reachable by CAF may have been fundamentally different than the overall population of adults with β-thalassemia in the USA. Patients involved with advocacy groups may have better connections to information, support, and healthcare, a better understanding of the disease, and other unobserved factors that may have contributed to a selection bias. The recruitment of patients who had received a blood transfusion in the previous month may have had an impact on patient-reported outcomes affected by variations in Hb levels [37], since the survey administration was relatively close to the last transfusion. If patients perceived overlap between different responses for time burden (such as time spent in waiting for blood to arrive versus time spent in the waiting room), certain time-related responses may have been inflated. The study results may have been influenced by the Coronavirus-2019 pandemic, as the data were collected between February and May 2021. Virtual business requirements and working from home may have impacted the patient-reported productivity burden, though this could not be explored since the nature of the patients’ jobs was not collected. Patients may have visited their healthcare providers less often during this time, and other factors related to healthcare and specific transfusion requirements may have been impacted. 

This study was not designed to differentiate outcomes between patients considered TDT or NTDT, or to explore the impact of genotype on outcomes, which would be of interest for future work. Clinical measures such as baseline Hb levels and treatment compliance were not collected, which may add context and insight to future research in this area. Finally, the sample size precluded exploration of subgroup analyses based on demographic and clinical characteristics of interest, which may have provided additional insight related to personalized care.

Since the current study is prospective in nature and has used data from CAF, a prospective long-term follow-up study that includes a nationally represented patient population is warranted to evaluate patient-reported outcomes before and after transfusions in patients with β-thalassemia.

## 5. Conclusions

This prospective study of patient-reported outcomes showed a substantial burden of β-thalassemia on fatigue, mental health, HRQoL, job decisions, WPAI, HCRU, and patient out-of-pocket costs. The time and financial requirements of a chronic RBC transfusion regimen were meaningful to a predominantly working-age population and their caregivers. In addition to improved therapeutic options, systemic strategies that reduce administrative requirements and waiting time, help remove clinical barriers to adherence, and support psychological and psychosocial well-being, could offer meaningful improvements to the QoL and clinical outcomes of patients with β-thalassemia and their families. 

## Figures and Tables

**Table 1:** Demographic and clinical characteristics, past 6 months.

Characteristic	Overall, *N* = 100
Age [mean ± SD], years	35.99 ± 10.36
Female, *n* (%)	65 (65)
Race, *n* (%)	
Asian or Pacific Islander	42 (42)
White	34 (34)
Hispanic	14 (14)
Mixed race	2 (2)
Other	7 (7)
Declined to answer	1 (1)
Marital status, *n* (%)	
Single, never married	49 (49)
Married/living with partner	46 (46)
Separated/divorced	5 (5)
Household income, *n* (%)	
USD < 15,000–24,999	27 (27)
USD 25,000–49,999	12 (12)
USD 50,000–99,999	15 (15)
USD ≥ 100,000	33 (33)
Declined to answer	13 (13)
Education, *n* (%)	
<High school graduate or equivalent (e.g., GED) to completed some school/college, but no degree	17 (17)
Associate degree/college graduate (e.g., B.A., B.S.)/completed some graduate school, but no degree	55 (55)
Completed graduate school (e.g., M.S., M.D., Ph.D.)	27 (27)
Declined to answer	1 (1)
Currently has health insurance, *n* (%)	98 (98)
Insurance type, *n* (%) ^a^	*N* = 98
Managed Care (HMO, PPO)	56 (57.1)
Medicaid (MediCal for California residents)/Medicare	46 (47)
High deductible health plan with HSA—also called “Consumer Directed Plan”	5 (5.1)
Other	11 (11.2)
Health insurance covers prescription medications, *n* (%)	89 (90.8)
Time since diagnosis [mean ± SD], years	34.40 ± 10.33
Prior treatment received for β-thalassemia, *n* (%)	
RBC transfusion	100 (100)
ICT	94 (94)
Surgery	47 (47)
Stem cell/bone marrow transplant	5 (5)
Other	3 (3)
Number of transfusions received in the past 6 months [mean ± SD]	9.62 ± 4.32
Frequency of transfusions, *n* (%)	
>1 per month	66 (66)
Monthly	31 (31)
Every other month	3 (3)
Time since last RBC transfusion, *n* (%)	
≤1 week	38 (38)
8–14 days	32 (32)
15–30 days	27 (27)
>1 month–4 months	3 (3)
RBC transfusion burden, *n* (%)	
Extremely high burden	14 (14)
High burden	20 (20)
Moderate burden	36 (36)
Some burden	21 (21)
No burden	9 (9)
Psychological burden Rating of β-thalassemia treatment routine (1—treatment has become routine, 7—treatment is a huge burden), *n* (%)	
1	41 (41)
2	24 (24)
3	10 (10)
4	9 (9)
5	6 (6)
6	5 (5)
7	5 (5)
Office visits, *n* (%)	92 (92)
Turned away for blood transfusion due to shortage, *n* (%)	10 (10)
Laboratory/blood test visits, *n* (%)	83 (83)
ER visits, *n* (%)	7 (7)
Hospitalizations, *n* (%)	3 (3)
Employed, *n* (%)	63 (63)
Employment decision affected by β-thalassemia, *n* (%)	52 (82.5)
How employment choice was affected by β-thalassemia, *n* (%) ^a^	
Chose for health insurance plan	37 (58.7)
Provided flexible hours	37 (58.7)
Provided needed job security	33 (52.4)
Not too physical	26 (41.3)
Close to a treatment center	18 (28.6)
Other reason	2 (3.2)

B.A.: Bachelor of Arts; B.S.: Bachelor of Science; ER: emergency room; GED: General Equivalency Diploma; HMO: health maintenance organization; HSA: Health Savings Account; ICT: iron chelation therapy; M.D.: Doctor of Medicine; M.S.: Master of Science; Ph.D: Doctor of Philosophy; PPO: preferred provider organization; RBC: red blood cell; SD: standard deviation. ^a^ Patients could select more than 1 response.

**Table 2:** Health-related quality of life outcomes.

HRQoL Parameters	Overall, *N* = 100
Depression symptom severity, *n* (%)	
None/minimal	48 (48)
Mild	24 (24)
Moderate	18 (18)
Moderately severe	7 (7)
Severe	3 (3)
Anxiety symptom severity, *n* (%)	
None/minimal	47 (47)
Mild	36 (36)
Moderate	12 (12)
Severe	5 (5)

HRQoL: health-related quality of life.

**Table 3:** Six-month time burden.

	Overall, *N* = 100 (Mean ± SD)
Number of HCPs seen for whole transfusion process (1 appointment)	5.11 ± 3.97
Number of follow-up visits in past 6 months (all patients)	
Treating HCPs	9.40 ± 8.16
Office visits	3.92 ± 3.75
Laboratory/blood test visits	6.09 ± 4.92
ER visits	0.10 ± 0.46
Hospitalizations	0.03 ± 0.17
Total time spent in the past 6 months, among patients reporting visits	
All office visits, hours (*n* = 92)	3.99 ± 5.41
All laboratory/blood test visits, hours (*n* = 83)	3.21 ± 2.85
All ER visits, hours (*n* = 7)	5.93 ± 3.45
Hospitalizations, days (*n* = 3)	2.17 ± 1.15
For treatment (e.g., scheduling, billing issues, insurance issues, etc.), hours	8.51 ± 11.41
Time spent in the past 6 months, hours	
Preparing for transfusion (i.e., getting blood drawn)	11.41 ± 28.28
Waiting for blood to arrive	41.60 ± 96.51
Waiting in the waiting room prior to the transfusion	15.32 ± 65.64
Getting the transfusion (being prepared and recovering)	80.92 ± 176.24
Traveling to and from the transfusion location	23.50 ± 51.49
Scheduling appointments	2.69 ± 2.84
Getting insurance verification	1.44 ± 2.97
Calling about billing issues	3.09 ± 5.40
Getting a/working with social worker	1.29 ± 3.79

ER: emergency room; HCP: healthcare professional; SD: standard deviation.

**Table 4:** Direct and indirect costs including 6-month costs.

	Overall, *N* = 100
Expenses outside of care, *n* (%)	
Childcare	9 (9)
Transportation	72 (72)
Parking/tolls	54 (54)
Prescription medication(s)	59 (59)
OTC (non-prescription) medication(s)	48 (48)
Other	8 (8)
Burden related to insurance issues, *n* (%)	
Extremely high burden	11 (11)
High burden	28 (28)
Moderate burden	15 (15)
Some burden	22 (22)
No burden	24 (24)
Costs in the past 6 months, USD	(*N* = 100)
Total OOPC related to care, among patients reporting visits, [mean ± SD], USD	896.15 ± 3066.84
Office visits (*n* = 92)	555.23 ± 1981.87
ER visits (*n* = 7)	143.00 ± 153.76
Hospitalizations (*n* = 3)	41.67 ± 52.04
Laboratory visits/blood tests (*n* = 83)	450.70 ± 1531.11
Amount of money spent for blood transfusion, [mean ± SD]	2239.18 ± 5537.33
Total costs outside of care, among patients reporting costs, [mean ± SD], USD	962.34 ± 2457.98
Childcare (*n* = 9)	1780.00 ± 2778.69
Transportation (*n* = 72)	178.83 ± 177.64
Parking/tolls (*n* = 54)	111.00 ± 131.50
Prescription medication(s) (*n* = 59)	791.59 ± 2501.01
OTC (non-prescription) medication(s) (*n* = 48)	157.5 ± 192.06
Other (*n* = 8)	885.00 ± 1584.53

ER: emergency room; OOPC: out-of-pocket costs; OTC: over-the-counter; SD: standard deviation.

## Data Availability

BMS policy on data sharing may be found at https://www.bms.com/researchers-and-partners/independent-research/data-sharing-request-process.html.

## Supplementary Materials

The following supporting information can be downloaded at: https://www.mdpi.com/article/10.3390/jcm12020414/s1. Table S1: Psychological burden and side effects of RBC transfusion, Table S2: Burden of ICT and comorbidities, Table S3: Caregiver burden.
