# Supplementary material for: Understanding the Association between Red Blood Cell Transfusion Utilization and Humanistic and Economic Burden in Patients with β-Thalassemia from the Patients’ Perspective
Source: J Clin Med. 2023 Jan 4;12(2):414. doi: 10.3390/jcm12020414 (PMC9861260; doi:10.3390/jcm12020414)
Supplement: Supplementary file 1 [file jcm-12-00414-s001.zip › jcm-2124693-supplementary/Supplementary Table S1_Revised.pdf]

Supplementary Table S1. Psychological burden and side effects of RBC transfusion.

	Overall, N = 100
**Side effects experienced during RBC transfusion, n (%)**	
Iron overload	81 (81)
Rash or hives	74 (74)
Fatigue	61 (61)
Pain or bruising at injection site	52 (52)
Fever	39 (39)
Headache	39 (39)
Severe allergic reaction	32 (32)
Bloating	32 (32)
Endocrine dysfunction	29 (29)
Trouble breathing	24 (24)
Stomach pain or discomfort/gastrointestinal issues	23 (23)
Dizziness	22 (22)
Liver dysfunction/damage	20 (20)
Chest pain	15 (15)
Kidney issues/nephropathy (a disease of the kidneys)	15 (15)
Other	12 (12)
Diabetes	11 (11)
Cardiovascular issues/heart failure	9 (9)
Alloimmunization	9 (9)
Bacterial contamination	6 (6)
Lung injury	3 (3)
**Concerns about fertility issues, among females, n (%)**	N = 65
Yes	37 (57)
No/Prefer not to answer	28 (43)
**Rating of acceptance of β-thalassemia treatment routine (1-acceptance of β-thalassemia, 7-frustration with β-thalassemia), n (%)**	
1	46 (46)
2	12 (12)
3	6 (6)
4	11 (11)
5	7 (7)
6	6 (6)
7	12 (12)
**Rating of wondering how life would be without β-thalassemia (1-wonders what life would be like w/o β-thalassemia, 7- does not wonder what life would be like w/o β-thalassemia), n (%)**	

1	34 (34)
2	26 (26)
3	11 (11)
4	9 (9)
5	7 (7)
6	6 (6)
7	7 (7)
**Rating of fatigue leading up to blood transfusion (1-never feels fatigued, 7-frequently feels fatigued), n (%)**	
1	1 (1)
2	6 (6)
3	5 (5)
4	2 (2)
5	16 (16)
6	24 (24)
7	46 (46)
**Rating of social life with friends and family related to blood transfusion schedule (1-not social, 7-social), n (%)**	
1	10 (10)
2	12 (12)
3	20 (20)
4	6 (6)
5	15 (15)
6	16 (16)
7	21 (21)

RBC: red blood cell.
