# Supplementary material for: Understanding the Association between Red Blood Cell Transfusion Utilization and Humanistic and Economic Burden in Patients with β-Thalassemia from the Patients’ Perspective
Source: J Clin Med. 2023 Jan 4;12(2):414. doi: 10.3390/jcm12020414 (PMC9861260; doi:10.3390/jcm12020414)
Supplement: Supplementary file 1 [file jcm-12-00414-s001.zip › jcm-2124693-supplementary/Supplementary Table S2_Revised.pdf]

Supplementary Table S2. Burden of ICT and comorbidities.

	**Overall, N = 100**
**Comorbidities (yes), n (%)**	
Anemia	33 (33)
Osteoporosis	33 (33)
Anxiety	18 (18)
Migraines/headache	11 (11)
Depression	10 (10)
Diabetes without complications	7 (7)
Liver disease, mild	7 (7)
Congestive heart failure	6 (6)
Liver disease, moderate or severe	5 (5)
Insomnia	5 (5)
Diabetes with complications	4 (4)
Melanoma	1 (1)
Leukemia	1 (1)
Acquired immune deficiency syndrome/Human immunodeficiency virus	1 (1)
Chronic pulmonary disease	1 (1)
Connective tissue disease	1 (1)
Heart attack	1 (1)
High blood pressure	1 (1)
Renal (kidney) disease, moderate or severe	1 (1)
Other (specify)	14 (14)
None of the above	29 (29)
**Charlson comorbidity index, n (%)**	
0	76 (76)
1–2	13 (13)
3+	11 (11)
**Received ICT, n (%)**	94 (94)
**Type of ICT received in past 6 months, n (%)**	
Oral	87 (93)
Subcutaneous pump	21 (22)
Intravenous	8 (9)
**Received more than one type of ICT, n (%)**	19 (19)
**Side effects experienced during ICT, n (%)**	
Nausea	38 (40)

Diarrhea	35 (37)
Abdominal pain	33 (35)
Pain or swelling at injection site	33 (35)
Hearing problems	28 (30)
Vomiting	19 (20)
Vision problems	19 (20)
Dizziness	18 (19)
Kidney or liver damage	16 (17)
None	15 (16)
Organ toxicity (build-up of chemicals in the organs)	13 (14)
Low blood pressure	12 (13)
Rash or hives	11 (12)
Fever	6 (6)
Hypocalcemia (low calcium levels in the blood)	4 (4)
Other	4 (4)
Severe allergic reaction	3 (3)
**Rating of burden of ICT (1 - acceptance of ICT, 7 - frustration with ICT), n (%)**	
1	10 (10)
2	12 (12)
3	20 (20)
4	6 (6)
5	15 (15)
6	16 (16)
7	21 (21)

ICT: iron chelation therapy.
