# Supplementary material for: Understanding the Association between Red Blood Cell Transfusion Utilization and Humanistic and Economic Burden in Patients with β-Thalassemia from the Patients’ Perspective
Source: J Clin Med. 2023 Jan 4;12(2):414. doi: 10.3390/jcm12020414 (PMC9861260; doi:10.3390/jcm12020414)
Supplement: Supplementary file 1 [file jcm-12-00414-s001.zip › jcm-2124693-supplementary/Supplementary Table S3_Revised.pdf]

Supplementary Table S3. Caregiver burden.

Caregiver parameters	Overall, N = 100
**Has a caregiver, n (%)**	13 (13)
**Amount of time caregiver spends per week, (mean ± SD), hours/week**	32.46 ± 37.32
**Relationship to caregiver, n (%)**	
Spouse or significant other	3 (23)
Parent	6 (46)
Sibling/friend/neighbor/other	4 (31)
**Live with caregiver, n (%)**	10 (77)
**Caregiver taking time off from work/school, n (%)**	
Yes, from work	6 (46)
Yes, from schooling/education	1 (8)
No	3 (23)
Does not apply	4 (31)
**Caregiver activities related to β-thalassemia (yes), n (%)**	
Help with household duties	10 (77)
Transportation	10 (77)
Assistance in treatment decisions	8 (62)
Picking-up prescription and over-the-counter (non-prescription) medications	8 (62)
Scheduling appointments	7 (54)
Assistance with personal hygiene	3 (23)
Assistance with childcare	1 (8)
**Impact on caregiver's physical health, n (%)**	
No negative impact at all	6 (46)
Not very negatively impacted	2 (15)
Somewhat negatively impacted	4 (31)
Very negatively impacted	1 (8)
Extremely negatively impacted	0
**Impact on caregiver's financial health, n (%)**	
No negative impact at all	4 (31)
Not very negatively impacted	2 (15)
Somewhat negatively impacted	5 (38)
Very negatively impacted	1 (8)
Extremely negatively impacted	1 (8)
**Impact on caregiver's mental/emotional health, n (%)**	
No negative impact at all	4 (31)

Not very negatively impacted	3 (23)
Somewhat negatively impacted	5 (39)
Very negatively impacted	0
Extremely negatively impacted	1 (8)
**Impact on caregiver's social health, n (%)**	
No negative impact at all	5 (39)
Not very negatively impacted	4 (31)
Somewhat negatively impacted	3 (23)
Very negatively impacted	1 (8)
Extremely negatively impacted	0

SD: standard deviation.
